# Ethanol consumption activates a subset of arcuate nucleus pro‐opiomelanocortin (POMC)‐producing neurons: a c‐fos immunohistochemistry study

**DOI:** 10.14814/phy2.15231

**Published:** 2022-03-21

**Authors:** Lauren E. Hood, Erin K. Nagy, Jonna M. Leyrer‐Jackson, M. Foster Olive

**Affiliations:** ^1^ Department of Psychology Arizona State University Tempe Arizona 85281 USA

**Keywords:** arcuate nucleus, c‐fos, ethanol, hypothalamus, POMC, pro‐opiomelanocortin

## Abstract

Ethanol activates various opioid peptide‐containing circuits within the brain that may underlie its motivational and rewarding effects. One component of this circuitry consists of neurons located in the arcuate nucleus (ArcN) of the hypothalamus which express pro‐opiomelanocortin (POMC), an opioid precursor peptide that is cleaved to form bioactive fragments including β‐endorphin and α‐melanocyte stimulating hormone. In this study, we sought to determine if ethanol intake activates ArcN POMC neurons as measured by expression of the immediate early gene c‐fos. Male and female POMC‐EGFP mice underwent drinking‐in‐the‐dark (DID) procedures for 3 consecutive days (2 h/day) and were allowed to consume either ethanol (20% v/v), saccharin (0.2% w/v), or water. On the fourth day of DID procedures, animals were allowed to consume their respective solutions for 20 min, and 1 h following the session brains were harvested and processed for c‐fos immunohistochemistry and co‐localization with EGFP. Our results indicate that ethanol intake activates a subset (~15–20%) of ArcN POMC neurons, whereas saccharin or water intake activates significantly fewer (~5–12%) of these neurons. The percent of activated POMC neurons did not correlate with blood ethanol levels at the time of tissue collection, and activation appeared to be distributed throughout the rostrocaudal axis of the ArcN. No sex differences were observed in the degree of neuronal activation across drinking solutions. These findings indicate a preferential activation of ArcN POMC neurons by ethanol consumption, strengthening the notion that ethanol activates endogenous opioid systems in the brain which may underlie its motivational properties.

## Graphical abstract

Ethanol consumption activates a subset of POMC neurons within the ArcN, which appear to be throughout most of the rostrocaudal axis. Saccharin and water consumption has lesser degrees of POMC neuronal activation.

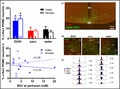

## 1 INTRODUCTION

Alcohol use disorders (AUDs), including those characterized by binge drinking, impose an enormous socioeconomic and medical burden worldwide. In the United States, costs of AUDs to society exceed $250B annually (Sacks et al., 2015). The endogenous opioid system has been extensively implicated in the rewarding, reinforcing, and neurochemical effects of ethanol (Gianoulakis, 2009; Herz, 1997; Roth‐Deri et al., 2008). The strongest evidence to date for such an involvement is the established clinical efficacy of the broad spectrum opioid receptor antagonist naltrexone in reducing ethanol intake, relapse propensity, and craving (Donoghue et al., 2015).

Endogenous opioid peptides that activate μ and δ opioid receptors (MOR and DOR, respectively), such as enkephalins and endorphins, possess intrinsic rewarding properties (Belluzzi & Stein, 1977; Goeders et al., 1984; Van Ree et al., 1979). Endorphins, including β‐endorphin, are derived from the pro‐opiomelanocortin (POMC) precursor peptide by proteolytic cleavage, a process which also results in the formation other POMC‐derived peptides such as adrenocorticotropic hormone (ACTH), alpha‐melanocyte‐stimulating hormone (α‐MSH), and β‐lipotropin (Cawley et al., 2016; Smyth, 2016). Cell bodies of POMC‐synthesizing neurons in the brain are largely confined to two distinct regions: the arcuate nucleus (ArcN) of the hypothalamus and the nucleus of the solitary tract (NTS) of the brainstem. Both regions give rise to widespread projections that innervate a variety of hypothalamic and extrahypothalamic regions of the brain, with some of degree of overlap. Major extrahypothalamic efferent targets of ArcN POMC neurons include the nucleus accumbens (NAc), bed nucleus of the stria terminalis, amygdala, paraventricular nucleus of the thalamus, periaqueductal gray, and various midbrain regions including the ventral tegmental area (VTA) (Finley et al., 1981; Gray et al., 1984; Jacobowitz & O'Donohue, 1978). Several of these regions, including the amygdala, are also innervated by POMC neurons originating in the NTS, although to a lesser degree (Khachaturian et al., 1985; Wang et al., 2015). ArcN POMC neurons also innervate other regions of the hypothalamus such as the zona incerta and the lateral, medial preoptic, dorsal and ventromedial, and posterior hypothalamic nuclei (Khachaturian et al., 1985; Wang et al., 2015).

Our laboratory and others have previously demonstrated that acute ethanol exposure in vivo induces the release of endorphin immunoreactivity in the NAc (Marinelli et al., 2003; Olive et al., 2001). Other laboratories have observed similar effects of ethanol in other regions including the ArcN, amygdala, and ventral midbrain (Jarjour et al., 2009; Marinelli et al., 2004). Such effects likely contribute to the motivational effects of ethanol, particularly in the amygdala, as MOR and DOR blockade in these regions reduces ethanol self‐administration (Foster et al., 2004; Hyytia & Kiianmaa, 2001). Recently, using patch clamp electrophysiology in brain slices of mice expressing enhanced green fluorescence protein (EGFP) under the control of the POMC promoter (POMC‐EGFP mice), we demonstrated that bath application of ethanol at low concentrations (5–40 mM) onto ArcN POMC neurons produces excitatory effects on a subset of these neurons, while in other subpopulations ethanol has either no effect or decreases POMC neuronal activity (Leyrer‐Jackson et al., 2020). However, whether these phenomena are observed following voluntary ethanol intake is not well studied.

The purpose of the present study was to examine if ethanol intake induces activation of ArcN POMC neurons as measured by immunohistochemistry for the activity‐dependent immediate early gene product c‐fos. To achieve this, we utilized POMC‐EGFP mice that were allowed to consume either ethanol, saccharin, or water using drinking‐in‐the‐dark (DID) procedures, an established model of ethanol consumption in rodents (Rhodes et al., 2005; Thiele et al., 2014). We also sought to determine whether the number of activated ArcN POMC neurons was correlated with blood ethanol levels, and whether anatomical differences could be observed in the patterns of activation across the rostrocaudal axis of the ArcN. To address this question, we utilized POMC‐EGFP mice which allow for accurate identification of POMC neurons based on their transgenic expression of EGFP. The use of these transgenic animals allows for minimizing any ambiguous labeling caused by the use of multiple antibodies during immunohistological staining, such as c‐fos and POMC used simultaneously.

## 2 METHODS

### 2.1 Animals

A total of *n* = 29 male and *n* = 34 female mice aged 3–8 months were used in the current study. Male POMC‐EGFP mice (C57BL/6J‐Tg(Pomc‐EGFP)1Low/J; (Cowley et al., 2001)) were obtained from Jackson Laboratories (Bar Harbor, ME; stock #009593) and bred with wild‐type female C57BL6J mice (Jackson Laboratories stock #000664) to produce heterozygous POMC‐EGFP mice. All mice were housed and bred in a temperature‐controlled vivarium (22–24°C) at Arizona State University. The colony room was set to a reverse light–dark cycle (14 h light/10 h dark, lights off at 07:00), as we found this light–dark cycle to be more conducive to breeding than standard 12:12 light–dark cycles. Mice were housed in standard polycarbonate cages and given standard rodent chow and water ad libitum except during DID testing where the water bottle was replaced with one of three experimental solutions (ethanol, saccharin or water) as described below. All experiments conducted were approved by the Institutional Animal Care and Use Committee at Arizona State University.

### 2.2 Genotyping

Tail snips were collected from animals between 4 and 6 weeks of age. The following primers for EGFP were utilized for genotyping and based on information provided by Jackson Laboratories: 5'‐ AAG‐TTC‐ATC‐TGC‐ACC‐ACC‐G (forward primer) and 5'‐TCC‐TTG‐AAG‐AAG‐ATG‐GTG‐CG (reverse primer). Using standard PCR‐based genotyping, all animals were confirmed to be positive for the EGFP transgene positive prior to experiments.

### 2.3 Drinking‐in‐the‐Dark (DID) procedures

At least 1 week prior to DID procedures, animals were placed into single housing conditions in standard polycarbonate cages. A 4‐day DID procedure was implemented for these studies (Rhodes et al., 2005; Thiele et al., 2014), whereby 3 h after lights off, water bottles were removed and replaced with a single 15 ml sipper tube (Drinko Measurer; Amuza Inc.) containing either 20% v/v ethanol, 0.2% w/v saccharin (as a palatable non‐caloric reference solution), or water as a control. Weights of the sipper tubes containing the solutions were measured before and after each session, and all mice were weighed prior to each DID session in order to assess ethanol consumption as a function of individual body weight. For the first 3 days, DID sessions were 2 h in duration. In our prior studies (Leyrer‐Jackson et al., 2021), we found that male mice consume most alcohol during the first 20 min of the DID session. Thus, on the fourth and final day, the length of the DID session was decreased to 20 min in order to capture neuronal activation that might occur due to initial drinking bouts. While this 20 min session does not give rise to intoxicating levels of consumption at the time of perfusion, this duration was chosen to capture the “load‐up” of alcohol consumption observed in our prior studies by male mice (Leyrer‐Jackson et al., 2021), and to avoid loss of the c‐fos expression caused by this load‐up consumption period that may occur with longer DID sessions. In addition, based on unpublished observations on blood alcohol clearance rates in POMC‐EGFP mice of approximately 30 mM per hour, we estimate that blood alcohol levels in these mice would be equivalent to an additional 30 mM above the levels we report in Figure 2b. However, analyses of actual blood alcohol levels following the 20 min DID session would be needed to confirm this. Furthermore, given that blood alcohol levels as well as c‐fos expression peak approximately one hour following consumption or neuronal activation, respectively, animals were not sacrificed until 60 min following their last DID session to capture fos activation at a timepoint reflective of peak blood alcohol levels. Following the last DID session (day 4), mice were asphyxiated with CO_2_ and upon lack of response to toe or tail pinch, the thoracic cavity was exposed. For animals consuming ethanol during DID procedures, a 40 μl blood sample was obtained from the heart, centrifuged at 1500*g* for 10 min, and the supernatant was stored at −20°C for subsequent analysis of blood ethanol concentration. Animals were then perfused with 20–50 ml of cold 1x phosphate‐buffered saline (PBS) at a speed of 3 ml/min, followed by 20–40 ml of 4% w/v paraformaldehyde. Following perfusion, brains were removed, post‐fixed in 4% paraformaldehyde for 24 h, and transferred to a 30% w/v sucrose cryoprotectant solution for at least 48 h.

### 2.4 Immunohistochemistry

Coronal sections throughout the rostrocaudal axis of the ArcN were collected on a cryostat at a thickness of 40 μm, washed in PBS 3 × 10 min, and placed into a blocking solution containing PBST (PBS +0.1% Triton X‐100) and 5% v/v normal donkey serum for 2 h at room temperature. Next, a rabbit anti‐c‐fos antibody (1:500; Cell Signaling Technology) was added to the blocking solution, and sections were then incubated overnight at 4°C under gentle agitation. On the next day, sections were washed 3 × 10 min in PBST, and an AlexaFluor568 conjugated donkey anti‐rabbit IgG secondary antisera (1:500; Abcam) was added for incubation for 2 h at room temperature. Finally, sections were washed 3 × 10 min in PBST followed by a 1 × 10 min wash in PBS, mounted onto microscope slides, coverslipped using Vectashield antifade mounting medium (Vector Laboratories), and sealed with clear nail polish.

### 2.5 Image acquisition and c‐fos quantification

Images were collected on a Leica SP5 confocal microscope from 8 to 10 randomly selected sections containing of the ArcN per mouse. Images were taken at 20x magnification and tiled to contain the entire ArcN. Excitation at 488 nm was used for visualization of EGFP, and excitation at 561 nm was used for visualization of c‐fos immunoreactivity. Quantification of c‐fos immunoreactivity was performed manually using the counting tool in ImageJ Fiji software by an investigator blind to experimental condition. Due to the variable number of POMC‐EGFP cells in each coronal section, the number of c‐fos immunoreactive cells showing co‐localization with EGFP was divided by the total number of EGFP positive cells to yield a percent of total EGFP cells expressing c‐fos. These percentage values then were averaged across all sections from each animal prior to being averaged within each sex and experimental group (ethanol, saccharin, or water). For assessment of the distribution of co‐localization of c‐fos immunoreactivity throughout the rostrocaudal axis of the ArcN, randomly selected sections from animals from each sex and experimental group were aligned to diagrams spanning from −1.34 to −2.70 mm posterior to bregma based on a stereotaxic atlas (Franklin & Paxinos, 2008), and the approximate location of c‐fos immunoreactivity and EGFP were noted.

### 2.6 Blood ethanol concentrations (BECs)

BECs were determined by an alcohol dehydrogenase based colorimetric assay (Kristoffersen & Smith‐Kielland, 2005). Each plasma supernatant sample was analyzed in duplicate. Assays were performed in 96‐well plates which were read on a plate reader at 340 nm absorbance. BECs were calculated from a polynomial equation generated by values from a standard curve (0–20 mM ethanol).

### 2.7 Data analysis

Data from *n* = 5 animals were excluded from consumption and immunohistochemical analyses due to unsatisfactory staining quality. Data from an additional *n* = 8 animals were excluded from correlational analyses due to either contamination of the blood sample (*n* = 3) or fading of c‐fos immunoreactivity (*n* = 5) during tissue storage; however, drinking data during the 4 DID sessions from these animals were included in the consumption analyses. Amount of ethanol (g/kg) or saccharin/water solution (ml) consumed during each DID session was analyzed using a mixed effects model, where session and sex were considered factors. Consumption data from the final (4th) DID session and percent of fos‐positive EGFP cells were each analyzed via two‐way ANOVA with sex and solution type as factors. Bonferroni post hoc comparisons were used to identify differences between groups. For correlations of c‐fos expression with BECs at time of perfusion, data from male and females were analyzed separately by simple linear regression. All statistical analyses were performed in GraphPad Prism 9.0, and *p *< 0.05 was considered statistically significant. All values are presented as the mean ± SEM (standard error of the mean).

## 3 RESULTS

### 3.1 Drinking‐in‐the‐dark procedures

**FIGURE 1:**
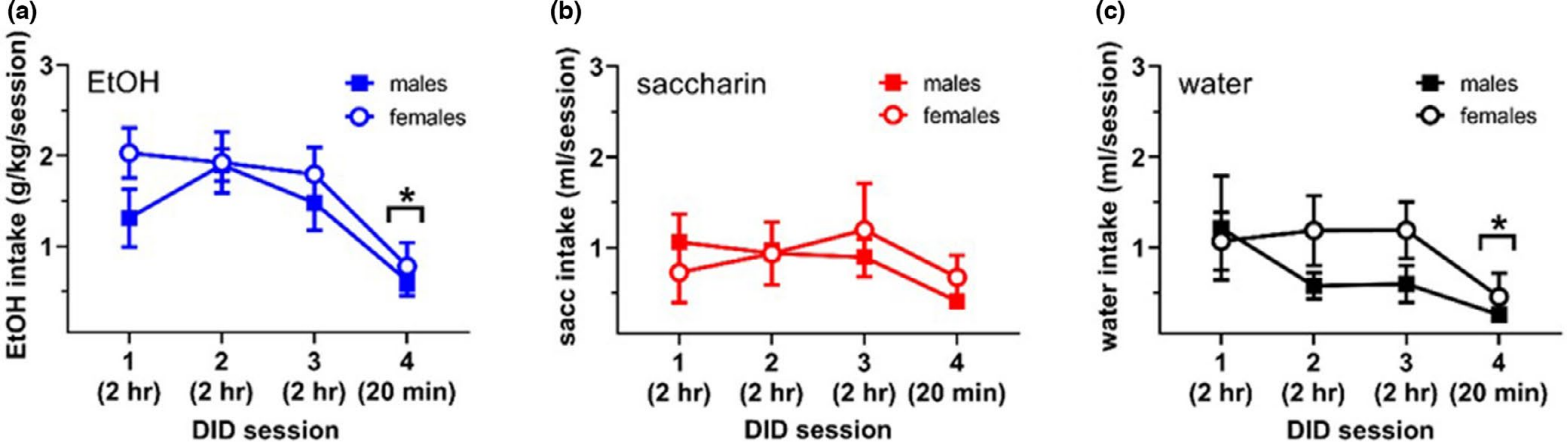
Amount of ethanol (EtOH, *a*), saccharin (*b*), and water (*c*) consumed during each of the 4 DID sessions. Group sizes for males are *n* = 10, *n* = 8, and *n* = 10 for panels *a*, *b*, and *c*, respectively. Group sizes for females are *n* = 13, *n* = 8, and *n* = 13 for panels *a*, *b*, and *c*, respectively. In panel *a*, * indicates *p *< 0.05 versus intake in sessions 1, 2, and 3 for both sexes. In panel *c*, * indicates *p *< 0.05 versus intake in session 3 for both sexes

Analysis of the amount of ethanol consumed across the four DID sessions revealed a significant effect of session (*F*_2,43_ = 18.57, *p *< 0.0001) but not sex (*F*_1,21_ = 1.56, *p *> 0.3205), nor an interaction between these factors (*F*_3,55_ = 1.92, *p *> 0.05). Bonferroni's post hoc multiple comparisons revealed that both sexes consumed significantly less ethanol during session 4 (20 min duration) as compared to sessions 1, 2 and 3 (2 h each in duration, Figure 1a). Analysis of the amount of saccharin solution consumed across the four DID sessions revealed no effect of session (*F*_2,21_ = 2.23, *p *> 0.05), sex (*F*_1,13_ = 0.03, *p *> 0.05) nor an interaction between these factors (*F*_3,39_ = 1.01, *p *> 0.05) (Figure 1b). Finally, analysis of the amount of water consumed across the four DID sessions revealed an effect of session (*F*_2,45_ = 4.21, *p *< 0.05), but not sex (*F*_1,21_ = 1.36, *p *> 0.05) nor an interaction between these factors (*F*_3,58_ = 1.26, *p *> 0.05). Bonferroni's post hoc multiple comparisons revealed that both sexes consumed significant less water during session 4 (20 min in duration) as compared to session 3 (2 h in duration, Figure 1c).

### 3.2 Co‐localization of c‐fos expression in POMC‐EGFP cells

**FIGURE 2:**
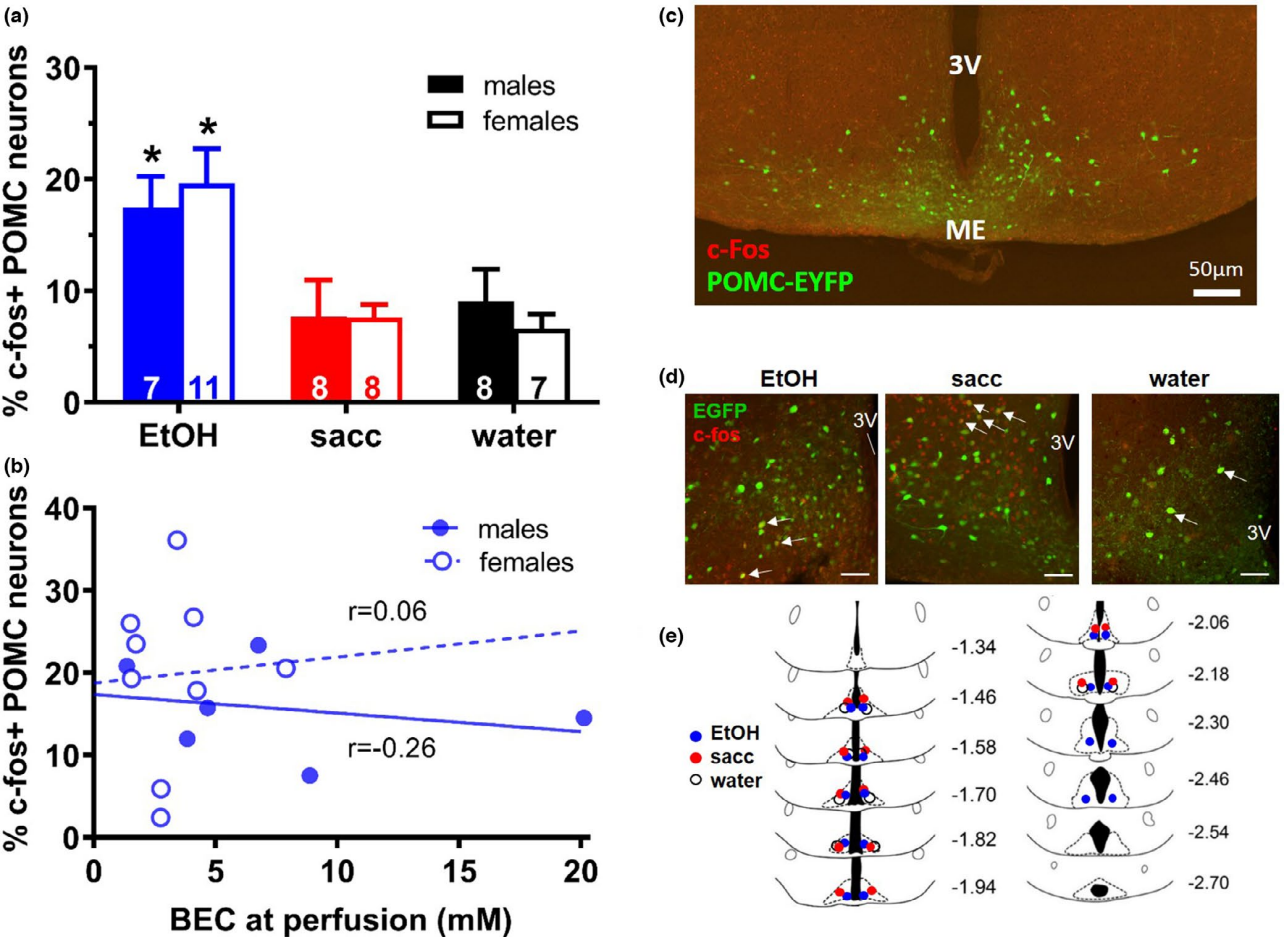
(a) The percentage of POMC‐EGFP cells in the ArcN that were positive for c‐fos immunoreactivity was greatest in both male and female mice consuming EtOH as compared to those consuming saccharin (sacc) or water (*p < 0.05). Group sizes are shown within vertical bars. (b) Lack of correlation between blood ethanol concentration (BEC) at the time of perfusion and the percentage of POMC‐EGFP cells in the ArcN that were positive for c‐fos immunoreactivity in either sex. *n* = 6 for males, *n* = 9 for females. (c) Representative low resolution photomicrograph of the ArcN containing EGFP positive neurons, c‐fos staining and major landmarks (3V, 3rd ventricle; ME, median eminence). (d) Representative images of c‐fos immunoreactivity co‐localized (arrows) to POMC‐EGFP cells in the ArcN from coronal sections in each experimental group of animals. Scale bars represent 50 μm. 3V, 3rd ventricle. (e) Approximate location of c‐fos immunoreactivity in POMC‐EGFP cells throughout the rostrocaudal axis of the ArcN for each group of animals. Numbers represent distance (in mm) of each section relative to bregma. Coronal drawing adapted from the atlas of Franklin and Paxinos (2008)

Due the variations in the number of POMC‐EGFP cells in the ArcN in each anatomical plane, we analyzed c‐fos immunoreactivity as a percent of the total number of POMC cells in each tissue section. A two‐way ANOVA, with solution type and sex as considered factors, revealed a significant effect of the type of solution (ethanol, saccharin, or water) consumed during the final DID session (*F*_2,41_ = 11.04, *p *< 0.0001), but no effect of sex (*F*_1,41_ = 0.001, *p *> 0.05) or an interaction between these factors (*F*_2,41_ = 0.38, *p *> 0.05). Bonferroni's post hoc multiple comparisons revealed that the percent of POMC‐EGFP neurons expressing c‐fos was significantly higher in animals consuming ethanol as compared to either saccharin or water (Figure 2a). However, we observed no significant correlation between the percent of POMC‐EGFP neurons expressing c‐fos and blood ethanol concentrations at the time of perfusion in either males (*r* = −0.26; *p *> 0.05) or females (*r* = 0.06, *p *> 0.05) (Figure 2b). A representative image of the ArcN containing POMC‐EGFP neurons and c‐fos immunolabeling is shown in Figure 2c. Representative images of c‐fos expression in POMC‐EGFP cells at −1.58 mm posterior to bregma are shown in Figure 2d for each group.

### 3.3 c‐Fos expression in POMC‐EGFP cells does not differ throughout the rostrocaudal axis

Next, in order to discern the approximate location of c‐fos immunoreactivity in POMC‐EGFP cells throughout the rostrocaudal axis of the ArcN, for each group we randomly selected immunostained sections and aligned them to corresponding coronal sections diagrams from the atlas of Franklin and Paxinos (2008). The percentage of cells showing co‐localization of c‐fos immunoreactivity with EGFP did not vary across the rostrocaudal axis (Figure 2e, *F*_1,10_ = 3.78, *p *> 0.05), with the exception that sections at the most rostral and caudal ends of the ArcN were generally devoid of c‐fos immunoreactivity and contained low numbers (< 10 per section) of POMC‐EGFP cells.

## 4 DISCUSSION

In the present study, we utilized POMC‐EGFP mice and c‐fos immunohistochemistry to demonstrate that ethanol consumption in a drinking‐in‐the‐dark (DID) paradigm results in activation of ~15–20% of POMC neurons in the ArcN of the hypothalamus. By comparison, animals consuming saccharin or water solutions in the same paradigm showed significantly less POMC neuronal activation (~5–12% of the total number of POMC‐EGFP cells). We also observed no differences between males and females in either overall fluid consumption patterns across 4 consecutive DID sessions, or in the number of activated POMC neurons within each consumption group. Finally, we did not observe any correlation between blood ethanol levels at the time of animal perfusion and the degree of activation of POMC neurons. These findings suggest that ethanol intake produces selective activation of a subset of POMC neurons in the ArcN of the hypothalamus.

In general, our observations are in agreement with several of our previous studies demonstrating activation of ArcN POMC neurons by ethanol. Using patch clamp electrophysiology in brain sections containing the ArcN from POMC‐EGFP mice, we showed that bath application of relatively low concentrations of ethanol (5–40 mM) produce increases in action potential firing in a subset of POMC neurons, while other cells from the same region showed either decreases or no change in firing patterns in response to ethanol (Leyrer‐Jackson et al., 2020). Also, using FosB as a marker of neuronal activation on combination with retrograding tracing, we observed that ethanol consumption (20% v/v) in the DID paradigm preferentially activates a subset ArcN POMC neurons that project to the amygdala, with little activation of these neurons projecting to the ventral tegmental area or nucleus accumbens (Leyrer‐Jackson et al., 2021). In this study, a similarly low percentage (~10%) of ArcN POMC neurons were activated by ethanol intake, which was greater than that activated by consumption of water. Indeed, most other studies examining the effects of ethanol, whether experimenter or self‐administered, on c‐fos expression in the rodent brain have primarily found activation in the paraventricular and medial preoptic areas of the hypothalamus, rather than the ArcN (reviewed elsewhere by (Vilpoux et al., 2009)). Thus, it is possible that the small subpopulation of ArcN POMC neurons activated by ethanol observed in the present set of experiments may have been overlooked in prior studies.

One possible reason for the relatively small number of ArcN POMC neurons activated by ethanol in the present study, as well as in our previous study (Leyrer‐Jackson et al., 2021; Leyrer‐Jackson et al., 2020) is the substantial molecular, anatomical, and function diversity of this population of neurons. On a functional level, ArcN POMC neurons have often been viewed as a homogeneous cell population that regulates food intake, glucose and lipid metabolism, and energy expenditure (Mercer et al., 2013; Quarta et al., 2021; Zhan, 2018). Typically, increased activity of ArcN POMC neurons is accompanied by decreases in feeding behaviors. However, a singularity of function of these neurons is likely to be overly simplistic, and is in fact in opposition to the findings of our current study, where we observed that consumption of a caloric solution (ethanol) produced evidence of increased POMC neuron activity as compared to intake of non‐caloric control solutions (saccharin or water). It is of note that in the current study, mice had access to chow throughout the DID sessions; however, quantification of chow consumption was not explored. Future studies examining whether changes in chow consumption occur across DID sessions or experimental groups are warranted for a clearer depiction of how POMC neuronal activity is altered in these circumstances. Given recent evidence for the possible role of ArcN POMC neurons in other processes such as nociception, cardiovascular function, fear and anxiety, locomotion, and reward (Quarta et al., 2021; Zhan, 2018), it is possible that these neurons may mediate ethanol reward and reinforcement independent of their ability to negatively regulate feeding behavior. However, the present study did not specifically compare the effects of ethanol intake with that of other palatable or caloric food substances on c‐fos immunoreactivity in ArcN POMC neurons, and this is an important avenue for further study.

On a molecular level, ArcN POMC neurons display a large degree of heterogeneity in terms of expression levels of genes encoding various receptors for classical neurotransmitters, neuropeptides, hormones, or transcription factors (Biglari et al., 2021; Campbell et al., 2017; Lam et al., 2017; Ma et al., 2021). In addition, ~50–75% of ArcN POMC neurons in the mouse are GABAergic, ~10–40% are glutamatergic, and up to 30% express both GABA and glutamatergic synthesizing enzymes (Hentges et al., 2009; Jarvie & Hentges, 2012; Stincic et al., 2018; Wittmann et al., 2013), suggesting dual excitatory and inhibitory functions. GABAergic POMC neurons tend to be distributed throughout rostrocaudal neuraxis of the ArcN, whereas glutamatergic POMC neurons tend to be more concentrated in caudal regions of the ArcN (van den Mercer et al., 2013; van den Pol et al., 2019). In the present study, we observed a relatively even distribution of POMC neurons co‐expressing c‐fos throughout the ArcN that were activated by either ethanol, saccharin or water intake, which is more in line with a GABAergic phenotype. However, this remains to be determined empirically, and it is of interest for future studies to explore the molecular profile of ArcN POMC neurons that are specifically activated by ethanol intake. Importantly, we have shown in prior studies that POMC neurons projecting to the amygdala are preferentially activated by ethanol, and these neurons likely display a distinct genetic profile compared to POMC neurons projecting elsewhere (Leyrer‐Jackson et al., 2021). This is especially important in light of our findings that the degree of ArcN POMC neuron activity was not correlated with blood ethanol levels at the time of tissue harvest. It is thus unknown what cellular mechanisms confer sensitivity to ethanol in subpopulations of these neurons, and whether excitation of these neurons by ethanol occurs locally within the ArcN or originates from modulation of excitatory or inhibitory inputs arising from other hypothalamic or extrahypothalamic regions.

We did not observe any sex differences in the present study with regards to levels of ethanol, saccharin or water intake during any of the DID procedures, nor in the percentage of activated ArcN POMC neurons as assessed by c‐fos immunohistochemistry. This was surprising, given that we previously observed that females drink more ethanol during DID procedures as compared to males (Leyrer‐Jackson et al., 2021), which has also been reported by others (Nentwig et al., 2019; Rhodes et al., 2007; Satta et al., 2018; Sneddon et al., 2019). Given that we saw robust sex differences in DID alcohol consumption in our previous study where females consumed more alcohol than males during sessions 2 and 3 utilizing a POMC‐cre‐tdTomato mouse line on a Lowl/B6 background, we hypothesized that we would also observe similar results utilizing POMC‐EGFP mice on a C57BL/6 background. However, such sex differences were not observed throughout sessions 1–3 in the current study. It is of note, that similar to our prior study, we did not observe sex differences within the utilized 20 min testing session, which allowed us to make direct comparisons between sexes regarding POMC activation (via c‐fos expression) without the confounding differences in levels of consumption. Interestingly, sex differences observed in other studies have been hypothesized to be driven, at least in part, by ovarian hormones (Radke et al., 2021; Satta et al., 2018). Specifically, a reduction of freely circulating hormones via ovary removal (ovariectomy) decreases alcohol consumption relative to ovary‐intact female mice (Satta et al., 2018). Additionally, treatment with the synthetic estrogen 17β‐estradiol‐3‐benzoate in ovariectomized mice restored drinking patterns to that of control (Satta et al., 2018), implicating estrogen levels play a pertinent role in driving intake in female mice. Furthermore, activation of both α‐ and β‐estrogen receptors within the hypothalamus can enhance dopaminergic signaling within the hypothalamus increasing output of the paraventricular nucleus and HPA axis responsivity in females (Finn, 2020; Rachdaoui & Sarkar, 2017). Thus through this mechanism, estrogen may promote higher drinking patterns in females due to increased levels of dopamine and glucocorticoids within the hypothalamus.

Interestingly, alterations in POMC associated genes, including POMC and β‐endorphin, have also been shown to modulate alcohol intake in a sex‐dependent manner. Specifically, deficiency in β‐endorphin has been shown to enhance voluntary binge‐like alcohol consumption in female but not male mice (Nentwig et al., 2019). However, it is of note that as observed in the current study Nentwig and colleagues did not observe sex differences in wild‐type mice with normal expressing levels of β‐endorphin (Nentwig et al., 2019). Furthermore, deficiencies in POMC utilizing the deletion of two neuronal POMC enhancers nPE1 and nPE2 decreased alcohol consumption in both male and female mice across a 4‐day DID paradigm utilizing 7.5, 15, and 30% concentrations of alcohol (Zhou et al., 2017). However, sex differences between POMC‐deficient mice were negligible, yet observed in wild‐type mice primarily at high alcohol concentrations (30%) (Zhou et al., 2017). Specifically, wild‐type females consumed more alcohol than their male counterparts during sessions 1 and 2 of DID but not sessions 3 and 4 (Zhou et al., 2017). In addition, some studies employing the DID paradigm have either not observed sex differences, or found that such differences do not emerge until after 10 DID sessions have been conducted (Hilderbrand & Lasek, 2018; Radke et al., 2021). Thus, the lack of observation of sex differences in the present study could be due to the fact that only 4 DID sessions were conducted. However, given that we and others have observed sex differences utilizing the same paradigm of only 4 DID sessions, this seems unlikely. Alternatively, another possible cause is the use of the POMC‐EGFP transgenic mouse strain, as there are strain differences known to exist with respect to patterns of intake of ethanol in the DID paradigm and/or two‐bottle choice procedures (Hilderbrand & Lasek, 2018; Radke et al., 2021; Rhodes et al., 2007; Yoneyama et al., 2008). Additionally, it is known that transgenic mouse strains can have unintentional neurotransmitter/peptide release and altered function of endogenous receptors (Chen et al., 2018; Chohan et al., 2020). One hypothesis, which has been unexamined in this transgenic line, is whether female mice of the POMC‐EGFP transgenic strain have altered levels of endogenous circulating estrogen levels or receptor expression levels on POMC neurons. A decrease in endogenous estrogen could result in normalizing consumption levels between the two sexes and thus, giving rise to the results reported here. However, future studies examining this hypothesis are warranted.

One limitation of the present study is what we did not determine either the neuropeptide phenotype (i.e., α‐MSH, β‐endorphin, ACTH, etc.) of neurons activated by either ethanol, saccharin, or water intake. It is therefore possible that each drinking solution type activated separate subpopulations of ArcN POMC neurons that synthesize different POMC‐derived neuropeptides, or that each solution type activated subpopulations with overlapping neuropeptide phenotypes. Similarly, we did not determine whether activated POMC neurons were GABAergic and/or glutamatergic in nature, nor their potential efferent target region(s). In our recent study, we demonstrated that of potential efferents projecting to the VTA, NAc or amygdala, ArcN POMC neurons activated by ethanol primarily project to the amygdala (Leyrer‐Jackson et al. 2021). It has also been demonstrated that acute ethanol exposure elevates in extracellular endorphin levels in the ventral midbrain, NAc, and amygdala (Jarjour et al., 2009; Marinelli, Quirion, & Gianoulakis, 2003, 2004; Olive et al., 2001), which may at least in part, be due to activation of ArcN POMC neurons. Additional studies are needed to further delineate the precise neurochemical identity and neural circuitry of POMC neurons activated by ethanol intake compared to that of other reinforcing substances.

In conclusion, the current study highlights that ethanol consumption activates ArcN POMC neurons to a greater extent that induced by saccharin or water solutions. However, only a subset of the total population of ArcN POMC neurons appear to be activated, further underscoring the molecular, cellular, and functional heterogeneity of ArcN POMC neurons. Despite this, we found that activation of these neurons by any of the three solutions consumed was consistent across the rostrocaudal axis, suggesting that ethanol does not have subregion‐specific effects within the ArcN. Given that ethanol intake and craving for ethanol are highly mediated by endogenous opioid peptides and their receptors, these data suggest that activation of subpopulations of ArcN POMC neurons may serve as neuroanatomical substrate for the rewarding and reinforcing effects of ethanol.

## CONFLICT OF INTEREST

None to declare.

## AUTHOR CONTRIBUTIONS

Lauren Hood, Erin Nagy, and Jonna Leyrer‐Jackson performed data collection, mouse breeding and genotyping, and contributed to manuscript edits and data interpretation; M. Foster Olive performed data collection and analyses, acquisition of images, prepared the manuscript, and handled revisions.

## Data Availability

All data will be made available upon request by the corresponding author.
